# Targeting GATA1 and *p2x7r* Locus Binding in Spinal Astrocytes Suppresses Chronic Visceral Pain by Promoting DNA Demethylation

**DOI:** 10.1007/s12264-021-00799-1

**Published:** 2021-12-10

**Authors:** Yan-Yan Wu, Hai-Long Zhang, Xiaomin Lu, Han Du, Yong-Chang Li, Ping-An Zhang, Guang-Yin Xu

**Affiliations:** 1 Jiangsu Key Laboratory of Neuropsychiatric Diseases, Institute of Neuroscience, Soochow University, Suzhou, 215123 China; 2 Department of Oncology, The Affiliated Haian Hospital of Nantong University, Nantong, 226600 China; 3 Center for Translational Medicine, The Affiliated Zhangjiagang Hospital of Soochow University, Suzhou, 215600 China

**Keywords:** Chronic visceral pain, GATA binding protein 1, Ten-eleven translocation 3, Purinergic receptor, Epigenetic regulation, Spinal astrocytes

## Abstract

Irritable bowel syndrome is a gastrointestinal disorder of unknown etiology characterized by widespread, chronic abdominal pain associated with altered bowel movements. Increasing amounts of evidence indicate that injury and inflammation during the neonatal period have long-term effects on tissue structure and function in the adult that may predispose to gastrointestinal diseases. In this study we aimed to investigate how the epigenetic regulation of DNA demethylation of the *p2x7r* locus guided by the transcription factor GATA binding protein 1 (GATA1) in spinal astrocytes affects chronic visceral pain in adult rats with neonatal colonic inflammation (NCI). The spinal GATA1 targeting to DNA demethylation of *p2x7r* locus in these rats was assessed by assessing GATA1 function with luciferase assay, chromatin immunoprecipitation, patch clamp, and interference *in vitro* and *in vivo*. In addition, a decoy oligodeoxynucleotide was designed and applied to determine the influence of GATA1 on the DNA methylation of a *p2x7r* CpG island. We showed that NCI caused the induction of GATA1, Ten-eleven translocation 3 (TET3), and purinergic receptors (P2X7Rs) in astrocytes of the spinal dorsal horn, and demonstrated that inhibiting these molecules markedly increased the pain threshold, inhibited the activation of astrocytes, and decreased the spinal sEPSC frequency. NCI also markedly demethylated the *p2x7r* locus in a manner dependent on the enhancement of both a GATA1–TET3 physical interaction and GATA1 binding at the *p2x7r* promoter. Importantly, we showed that demethylation of the *p2x7r* locus (and the attendant increase in P2X7R expression) was reversed upon knockdown of GATA1 or TET3 expression, and demonstrated that a decoy oligodeoxynucleotide that selectively blocked the GATA1 binding site increased the methylation of a CpG island in the *p2x7r* promoter. These results demonstrate that chronic visceral pain is mediated synergistically by GATA1 and TET3 *via* a DNA-demethylation mechanism that controls *p2x7r* transcription in spinal dorsal horn astrocytes, and provide a potential therapeutic strategy by targeting GATA1 and *p2x7r* locus binding.

**Supplementary Information:**

The online version contains supplementary material available at 10.1007/s12264-021-00799-1.

## Introduction

Irritable bowel syndrome (IBS)-like chronic visceral pain symptoms are now reported by 25% of the population, yet our scientific understanding of the neurophysiological basis of this constellation of disorders is quite limited. It was believed that adverse stimulation (such as injury and inflammation) during developmental periods might contribute to nervous system diseases including chronic visceral pain in adults, yet the mechanism remains unclear. The impact of DNA methylation in regulating gene transcription is now understood to be central to the maintenance of normal body functions [1, 2]. Disruption of DNA methylation affects many disorders including developmental diseases, inflammation, and chronic pain [3, 4]. Emerging evidence has shown that both DNA methylation and demethylation are involved in nociceptive and anti-nociceptive processes in the peripheral and central nervous systems [5, 6]. Other studies have suggested that the promotion of interactions between transcription factors and demethylated gene promoters in the peripheral nervous system impacts neuropathic pain and gastric hyperalgesia [7, 8]. However, the relationship between the selective binding of transcription factors and the regulation of DNA methylation in visceral pain remains unknown.

GATA1 is a prototypical lineage-restricted transcription factor that functions to regulate the normal differentiation, proliferation, and apoptosis of erythroid and megakaryocytic cells, doing so through its cooperative regulation of key molecules like BCL­X_L_ and FOG1 [9, 10]. In addition, up-regulation of GATA1 has been reported to negatively regulate synaptic genes, and GATA1 up-regulation has been associated with major depression disorder [11, 12]. However, the regulatory mechanism(s) mediated by GATA1 remain unclear. Notably, GATA1 has been reported to participate in the precise regulation of gene expression as cells pass through mitosis [13], and the selective binding of GATA1 is known to be mediated by the histone modification H3K4me1 [14], indicating the involvement of GATA1 in epigenetic regulatory processes. Consistent with this, a recent study reported that alterations in chromatin, including H3K27me3, are involved in the GATA1-mutation-related pathogenesis of Diamond-Blackfan anemia [15]. However, any interactions through which GATA1 may impact DNA demethylation remain unknown.

Studies using rat models of IBS have shown sensitization of purinergic P2X receptors (P2XRs) in dorsal root ganglion (DRG) neurons [16], indicating a role for peripheral P2X in chronic visceral pain. Notably, P2X7Rs have several distinguishing features compared to other subtypes of purinergic receptors [17–19], and P2X7Rs have been shown to impact many aspects of neuronal physiology and neuronal pathology, including neurotransmitter release, neuroinflammation, neurodegenerative diseases, and neuropathic pain [19–21]. P2X7Rs have also been reported to play a role in intestinal inflammation, and this protein is known to trigger the development of chronic visceral pain [22]. More recently, neonatal maternal deprivation has been shown to increase P2X7R accumulation and to enhance presynaptic transmission in the insular cortex of an adult rat model of chronic visceral pain [23]; however, any regulatory mechanism(s) underlying this hyperalgesia-related accumulation remain unclear.

In the present study, we explored the interaction mechanism of GATA1, P2X7R and hydroxymethylase TET3 in the occurrence and development of chronic visceral pain in the NCI-induced rat model of chronic visceral pain.

## Materials and Methods

### Induction of Chronic Visceral Pain

Experiments were performed on male Sprague-Dawley rats (180 ± 20 g). To avoid the periodic effects of estrogen on pain [24], we studied male rats only to ensure the uniformity and repeatability of experiments. Care and handling of these animals were approved by the Institutional Animal Care and Use Committee of the Soochow University and were in accordance with the guidelines of the International Association for the Study of Pain. Chronic visceral pain was induced in rats by neonatal colonic inflammation (NCI). Briefly, 10-day-old pups received an infusion of 200 μL of 0.5% acetic acid in saline into the colon (2 cm from the anus). Controls received an equal volume of normal saline (NS).

### Measurement of Chronic Visceral Pain

Chronic visceral pain was assessed by the colorectal distention (CRD) threshold as described in previous studies [25]. In detail, a flexible balloon made of a surgical glove finger (length, 6 cm; diameter, 2 cm) was attached to a polyethylene tube. Rats were lightly sedated with isoflurane while the tube was inserted 8 cm into the descending colon and rectum *via* the anus. The tube was then held in place by taping the tube to the tail. After the rats were allowed to recover in a small and separate clear box for 30 min, CRD was performed. By quickly inflating the balloon until an obvious abdominal withdrawal reflex occurred, the CRD threshold was recorded (in mmHg) using a sphygmomanometer in a blinded manner. Measurement were repeated three times, every two minutes, and the average CRD threshold was calculated. Experiments were performed on these rats at 6 weeks of age.

### Drug Administration

A438079, a known selective inhibitor of the P2X7R, was purchased from Tocris (Bristol, UK) and freshly prepared in DMSO (Sigma, St Louis, MO, USA) before use. For the behavioral measures, a single intrathecal injection of different doses (10, 30, or 100 μg in 10 μL DMSO) of A438079 was administered. Briefly, at the age of 5 weeks, a guiding needle (18 G) was passed between lumbar vertebrae 5 and 6 to enter the intrathecal space and the A438079 solution was slowly injected. To determine the long-time effect, A438079 was administered intrathecally at 30 μg in 10 μL daily for 7 consecutive days. Tissues from these rats were used for molecular expression experiments.

To verify the role of TET3 and GATA1, we used their specific siRNAs (from RiboBio, Guangzhou, China). Before intrathecal injection, 5 nmol siRNA and negative control-siRNA were each dissolved in 100 μL deionized, diethylpyrocarbonate-treated water. To further examine the long-term effects, the siRNA (0.5 nmol/10 μL) was intrathecally injected once a day for consecutive 7 days. CRD thresholds were recorded beginning on day 8 at 0.5, 1, 2, 4, 8, 12, 24, 48, 72, and 96 h after siRNA treatment.

Fluorocitrate from Sigma (St Louis, Missouri, USA) was used to inhibit the activation of astrocytes. When delivered, 5 mg fluorocitrate was dissolved in 100 mL DMSO then stored at –20°C. For the behavioral measures, a single intrathecal injection of different doses (0.5, 1, or 2 nmol diluted in 10 μL DMSO) was used.

A decoy oligodeoxynucleotide (ODN) was designed to block the binding site of GATA1 at the *p2x7r* promoter. After synthesis by Sangon Biotech Co., Ltd (Shanghai, China), the decoy ODN was dissolved in normal saline. To examine the long-time effects, decoy ODN (20 μmol/L in 10 μL) was intrathecally injected once a day for consecutive 7 days. Tissues from these rats were used for molecular investigation experiments.

### Immunofluorescence Study

The rats were deeply anesthetized and perfused transcardially with 300 mL 0.9% normal saline followed by precooled 4% paraformaldehyde (PFA). The T13-L2 spinal cord segments were rapidly removed, post-fixed in PFA for 3 h, and then dehydrated in a gradient of phosphate-buffered sucrose. For double labeling, 14 μm sections of spinal cord were incubated with the primary antibody against GATA1 (1:200, Cell Signaling Technology, 3535), P2X7R (1:100, Alomone labs, APR004), TET2 (1:200, Merck Millipore, ABE364), or TET3 (1:50, Santa Cruz, sc-139186), together with the cell marker antibody against NeuN (1:50, Merckmillipore, MAB377), GFAP (1:300, Cell Signaling Technology, 3670S), or CD11b (1:100, Bio-Rad, MCA275R) at 4°C overnight and then incubated with Alexa Fluor 488 (1:500, Molecular Probes, A21206) and 555 (1:100, Molecular Probes, A31570) for 2 h at room temperature. Negative controls were obtained by omitting the primary antibodies.

### Western Blotting

SDS-PAGE and immunoblotting were applied as we previously described [23]. Briefly, 10% polyacrylamide gels were used to detect GATA1 (1:1000, Cell Signaling Technology, 3535) P2X1R (1:500, Alomone labs, APR001), P2X2R (1:500, Alomone labs, APR003), P2X3R (1:500, Alomone labs, APR016), P2X4 (1:500, Alomone labs, APR002), P2X7R (1:500, Alomone labs, APR004), TET1 (1:100, Santa Cruz, sc-163443), TET2 (1:1000, Merck Millipore, ABE364), and TET3 (1:100, Santa Cruz, sc-139186). GAPDH (1:1000, Hangzhou Goodhere Biotechnology, AB-P-R001) served as the internal control. Bands were visualized using an enhanced chemiluminescence detection kit for HRP (EZ-ECL, Biological Industries 20-500-120) and appropriate exposure to the chemiluminescent imaging system (ChemiDoc XRS, Bio-Rad, Hercules, CA, USA). Band intensities were measured using ImageJ software. Protein expression was normalized to GAPDH.

### Real-time Quantitative Polymerase Chain Reaction (qPCR)

Total RNA was exacted from the T13-L2 dorsal horn from control and NCI rats by the TRIzol method. cDNA was synthesized from total RNA using an EasyScript First-Strand cDNA Synthesis SuperMix kit (Transgen Biotech, Beijing, China) following the instructions. The primer sequences used in qPCR are listed in Table 1. Negative control reactions were performed by omitting the cDNA template. The relative expression level for each target gene was normalized *via* the 2^-ΔΔCt^ method.

**Table 1:** Primer sequences used in qPCR assay

Primers	Sequences (5′ to 3′)
P2X7R-F	CGGCACCATCAAGTGGATCTT
P2X7R-R	CTGCAACGCCTTTGACCTTG
GATA1-F	CCTGTCACCAGCAGTGCTTA
GATA1-R	ACTCTCTGGCCTCACAAGGA
DNMT1-F	TACGCAAGGTTTGAGTCCCC
DNMT1-R	CCCAGTCGGTAGACAACACC
DNMT3a-F	GAGGGAACTGAGACCCCAC
DNMT3a-R	CTGGAAGGTGAGTCTTGGCA
DNMT3b-F	CATAAGTCGAAGGTGCGTCGT
DNMT3b-R	ACTTTTGTTCTCGCGTCTCCT
TGFα-F	GCTGATCCACTGCTGTCAGG
TGFα-R	CAGGCAGTCCTTCCTTTCAGG
CNTF-F	GGAATCTTATGTAAAACATCAGGGC
CNTF-R	TTGCCACTGGTACACCATCC
IL6-F	GCAAGAGACTTCCAGCCAGT
IL6-R	TTGCCATTGCACAACTCTTTTCT
OSM-F	ACACTGCTTAGTTTGGCCCT
OSM-R	CGTGATGTTCGCCTGACTCT
LIF-F	GATTGTGCCCCTACTGCTCA
LIF-R	CCCCTTGAGCTGTGTAATAGGAAA
GAPDH-F	TGGAGTCTACTGGCGTCTT
GAPDH-R	TGTCATATTTCTCGTGGTTCA

### Methylation-specific PCR (MSP) and Bisulfite Sequencing

Two CpG islands in the P2X7R promoter were predicted by MethPrimer online. MSP was used following the supplier’s instructions. Briefly, genomic DNA was extracted from the spinal dorsal horn of control and NCI rats using a QIAamp DNA Mini Kit (Qiagen, Valencia, CA) and then modified with an EpiTect Bisulfite Kit (QIAGEN, Valencia, CA) following the manufacturer’s instructions. After that, modified DNA was used in MSP amplification. The MSP specific primer sequences were designed as follows: methylated primer forward (5′-ATTTTAGTAAGTTAGAGGTGGAGGC-3′) and reverse (5′-TTCAAACAAAAACCTATCAAACGTA-3′); unmethylated primer forward (5′-TTTT-AGTAAGTTAGAGGTGGAGGTG-3′) and reverse (5′-TTCA- AACAAAAACCTATCAAACATA-3′).

The methylation status was further validated by bisulfite sequencing. The following primers were designed to amplify the CpG2 island regions within P2X7R: forward (5′-AATTTTAGTAAGTTAGAGGTGGAGG-3′) and reverse (5′-TAATT- CAAACAAAAACCTATCAAAC-3′).

### Chromatin Immunoprecipitation (ChIP) Assay

A ChIP assay was performed using EZ-Magna ChIP A/G (Millipore, Charlottesville, VA) according to the supplier’s protocols. In brief, the spinal dorsal horns from control and NCI rats were separately lysed and sonicated 6–8 times in an ice bath. The input control was 10% of the lysate, and the remaining 90% was incubated with anti-GATA1 (1:50, Santa Cruz, sc-265 X) antibody or rabbit IgG overnight at 4°C. The immunoprecipitated complexes were collected by Magna GrIP™ Rack (Millipore, Charlottesville, VA). Then the precipitates were washed and incubated with ChIP wash buffer and Proteinase K (Millipore, Charlottesville, VA) at 62°C for 2 h. After that, the DNA was purified using columns supplied with the EZ-Magna ChIP A/G kit. Then, the extracted DNA was subjected to PCR amplification using the GATA1 binding site-specific primers forward (5′-CAAAAGAAACGGATGGGTCTGAG-3′) and reverse (5′-TGATTCAGACAAGGGCCTATCAG-3′). The amplification product was separated by 2% agarose gel electrophoresis.

### Luciferase Reporter Gene Assay

First, the recombinant plasmid of GATA1 and luciferase reporter gene were constructed as follows: the total RNA from the spinal dorsal horn of rats was exacted with TRIzol (Ambion, Shanghai, China). cDNA synthetized by an RT kit (Transgen Biotech, Beijing, China) was subjected to PCR amplification with the GATA1 primers (forward 5′-CGCTCTAGCCCGGGCGGATCCATGGATTTTCCT-GGTCTAGGGG-3′; reverse 5′-AGACTCGAGAGATCTGTCGACTCAAGAACT-GAGTGGAGACACTACG-3′). The amplification products were connected to the pCMV-C plasmid (Beyotime, Nantong, China) using ClonExpress II One Step Cloning Kit (Vazyme, Nanjing, China) following the manufacturer’s protocol.

The whole genomic DNA was separated from the spinal dorsal horn according to the instructions with the QIAamp DNA Mini Kit (Qiagen, Valencia, CA). After that, the total DNA was subjected to PCR amplification using the P2X7R CpG2 island-specific primers (forward 5′-CAGAACATTTCTCTGGCCTAAC-TGGCAAATGTGAAGTCAGCCTGGTTGC-3′; reverse 5′-TCTACGCGTGAGC-TCCTCGAGAAGGGCCTATCAGACGTGTTCG-3′). The amplification products were connected to the pGL6-TA reporter gene plasmid (Beyotime, Nantong, China) following the instructions with the ClonExpress II One Step Cloning Kit (Vazyme, Nanjing, China).

The recombinant plasmids were transfected into HEK293T cells as follows: (1) *p2x7r-pGL6* plasmid alone; (2) *p2x7r-pGL6* plasmid together with *gata1-pCMV* plasmid. Luciferase assays were performed 48 h after transfection using the Firefly Luciferase Reporter Gene Assay Kit (Beyotime, Nantong, China) according to the supplier’s protocol.

### Patch Clamp Recording from Spinal Cord Slices

Patch clamp recordings were made as previously reported [25]. After laminectomy, the dorsal aspect of the vertebral column was exposed, and the T13-L2 lumbar region was removed and immersed in ice-cold sucrose-based artificial cerebral spinal fluid (SACSF) saturated with 95% O_2_/5% CO_2_. The SACSF contained (in mmol/L): 50 sucrose, 95 NaCl, 1.8 KCl, 1.2 NaH_2_PO_4_, 7 MgSO_4_, 0.5 CaCl_2_, 26 NaHCO_3_, and 15 Glucose. The colon-related part of the spinal cord was exposed and carefully removed with the T13-L2 segment dorsal roots attached. The spinal cord was rapidly removed and embedded in 3% high strength agarose (type I-B, Sigma, USA). Spinal cord slices (450 μm) were cut with the Vibroslicer VT1200 S (Leica, Germany) and transferred to 31°C SACSF to recover for 30 min and kept for the next 4–5 h. The slices were then transferred to the recording chamber and continuously perfused with recording ACSF with the following composition (in mmol/L): 127 NaCl, 1.8 KCl, 1.2 NaH_2_PO_4_, 2.4 CaCl_2_, 1.3 MgSO_4_, 26 NaHCO_3_, and 15 Glucose. The perfusion rate was ~2 mL/min. The large lamina II neurons consistent with projection neurons were selected for recording. Each new experimental protocol used a fresh slice.

Neurons used for recording in lamina II were visualized using infrared differential interference contrast video microscopy with a 40× magnification water-immersion objective (BX51WI, Olympus, Shinjuku-ku, Tokyo, Japan). Patch electrodes (4–8 MΩ tip resistance) were made from borosilicate glass capillaries using a Flaming/Brown P-97 micropipette puller (Sutter Instruments Co). The internal solution for recording excitatory post-synaptic currents (EPSCs) and action potentials (APs) contained (in mmol/L): 140 K-Gluconate, 4 NaCl, 0.2 EGTA, 10 HEPES, 2 Mg-ATP, and 0.3 Na-GTP, pH adjusted to 7.2–7.3 with KOH. After gigaohm (GΩ) seals (usually >1 GΩ) were formed and the whole-cell configuration was obtained, neurons were tested if the resting membrane potential was more negative than −50 mV and direct depolarizing current injections evoked APs overshooting 0 mV when recording EPSCs and APs. We only included data from excitatory neurons according to the electrophysiological characteristics described by Washburn and Moises in response to intracellular injection of a depolarizing current (100–300 pA, 50-pA step, 1000-ms duration) in the further analyses of spontaneous (s)EPSCs. The holding potential was −70 mV for recording EPSCs. All drugs were dissolved in recording ACSF on the day of the experiment and applied by perfusion.

Data were acquired using a Digidata 1440A interface, MultiClamp 700B amplifier, and pClamp10 software. Data were sampled and filtered at 10 kHz with a Bessel filter in the amplifier. To ensure high-quality recordings, series resistance (<20 MΩ) was checked using the membrane test function of the pClamp10 software throughout the experiment. Data were stored on a computer and analyzed offline.

### Statistical Analyses

All values are shown as the mean ± standard error. Statistical analyses were done using Prism 7 (Graph Pad, San Diego, CA, USA) and OriginPro 8 (OriginLab, Northampton, MA) software. The Gaussian distribution test was applied before analysis. The two sample *t*-test or Mann-Whitney test was used to determine the significance of differences between two groups. Two-way ANOVA followed by Tukey’s *post hoc* test or one-way ANOVA analyses followed by Dunnett’s test was used when appropriate. *P* < 0.05 was considered statistically significant.

## Results

### Activation of Spinal Astrocytes Contributes to Chronic Visceral Pain

**Fig. 1:**
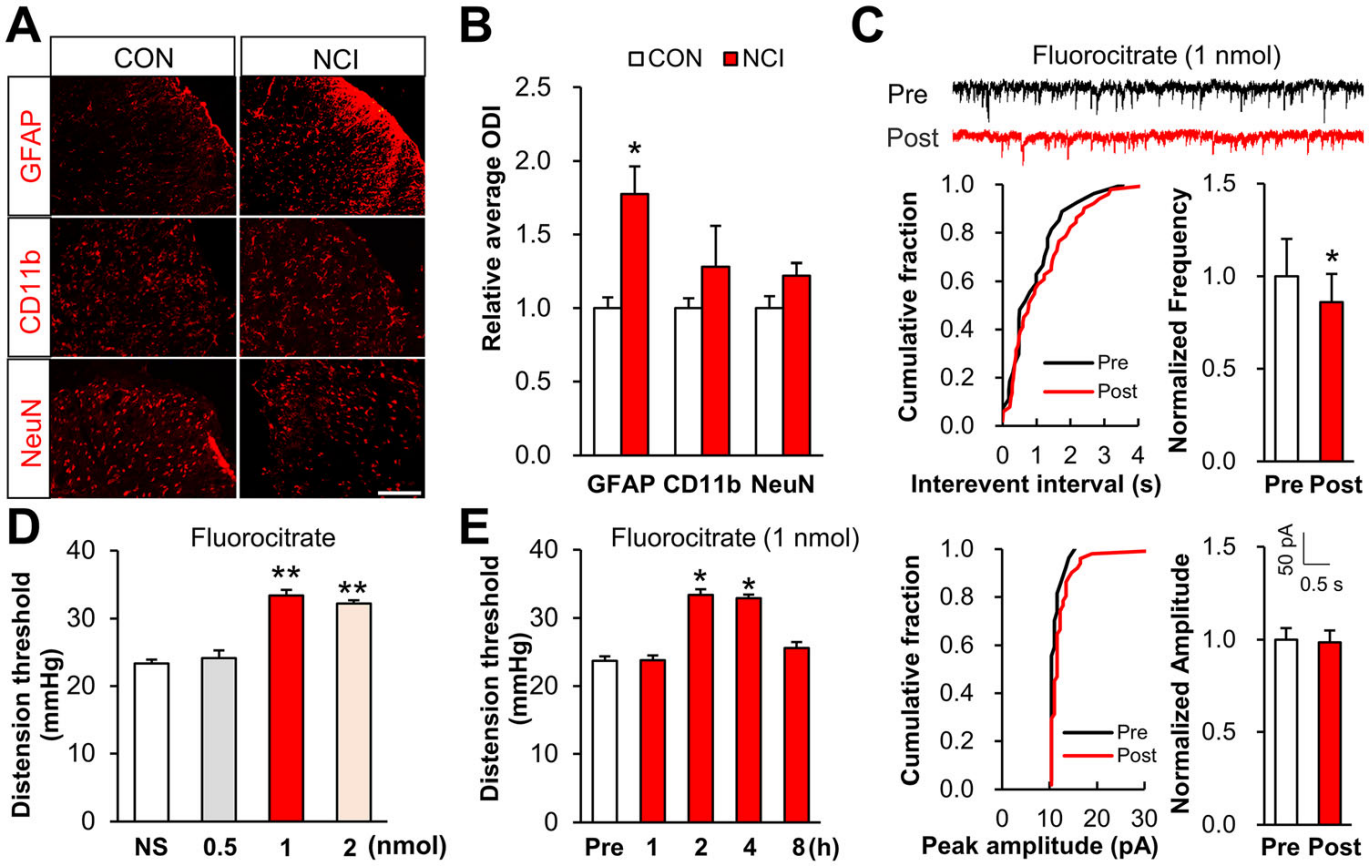
Activation of spinal astrocytes contributes to chronic visceral pain of NCI rats. **A, B** The number of GFAP-labeled astrocytes is increased in the spinal dorsal horn of NCI rats (scale bar, 100 μm, *n* = 4 per group, *P < 0.05 *vs* CON, two sample *t*-test). **C** Incubation with fluorocitrate (1 μmol/L, astrocyte inhibitor) markedly reduces the sEPSC frequency but does not alter sEPSC amplitude (*n* = 8 for fluorocitrate treatment, *P < 0.05 *vs* Pre, two sample *t*-test). **D, E** Intrathecal injection of fluorocitrate reverses the CRD threshold at 1 or 2 nmol in 10 μL; this reversion lasts for ~4 h (NS: normal saline, *n* = 7 for NS group, *n* = 6 for 0.5 nmol group, *n* = 7 for 1 nmol group, *n* = 5 for 2 nmol group, *P < 0.05 *vs* Pre, **P < 0.01 *vs* NS, one-way ANOVA followed by Dunnett’s tests).

Chronic visceral pain was induced by NCI and measured as the CRD threshold from control and NCI rats as previously reported. Immunofluorescence analyses were performed to investigate the spinal mechanism of chronic visceral pain in NCI rats. Compared to control animals, the NCI rats exhibited clear activation of spinal astrocytes; no activation of microglia or neurons was detected (Fig. 1A, B). Chemical inhibition assays in which astrocytes of NCI rats were treated with the known astrocyte activation inhibitor fluorocitrate revealed that the frequency but not the amplitude of sEPSCs was markedly decreased (Fig. 1C). Importantly, the application of the fluorocitrate to NCI rats reversed the CRD threshold, doing so in a dose- and time-dependent manner. Fluorocitrate treatment at 0.5 nmol had no significant effect on the CRD threshold of NCI rats; however, when the dose was increased to 1 nmol or 2 nmol, the threshold was remarkably increased (Fig. 1D). This reversal effect was sustained for at least 4 h after intrathecal fluorocitrate injection (1 nmol, Fig. 1E). Collectively, these results show that spinal astrocytes contribute to increased spinal synaptic transmission, thereby helping explain the increased chronic visceral pain in the NCI model rats.

### P2X7R Expression is Elevated in Spinal Dorsal Horn Astrocytes of NCI Rats

**Fig. 2:**
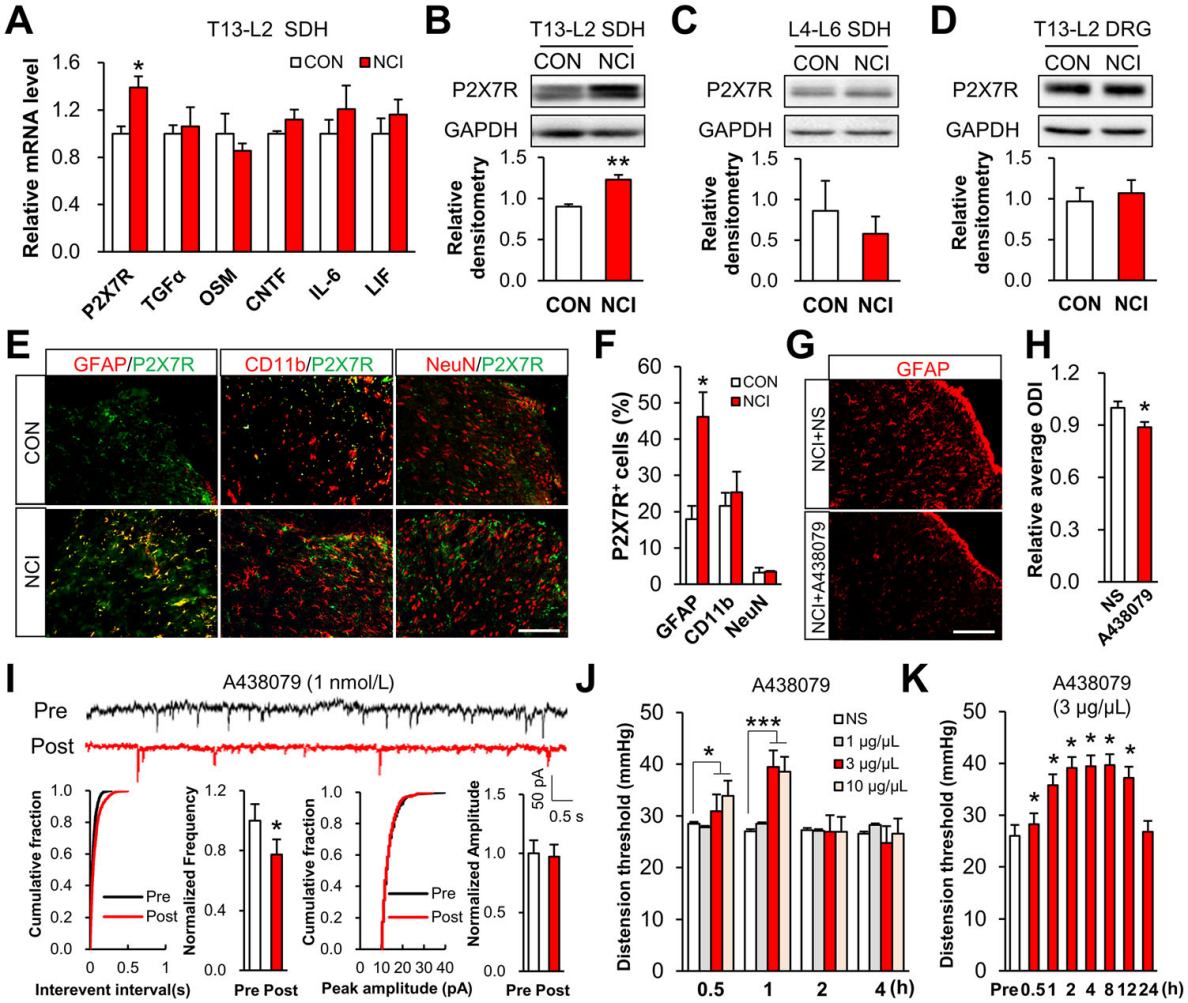
Accumulation of P2X7R in spinal dorsal horn astrocytes leads to chronic visceral pain in NCI rats. **A, B** QPCR and Western blotting analyses of the mRNA and protein levels of P2X7R reveal significant elevation, specifically in spinal dorsal horn astrocytes of 6-week-old NCI rats. Note that there is no difference between NCI model and control rats in the mRNA levels of *TGFα, OSM, CNTF, IL-6, or LIF* (*n* = 4 per group, *P < 0.05, **P < 0.01 *vs* CON, two sample *t*-test). **C, D** There is no difference in the P2X7R levels in the L4-6 spinal cord or T13-L2 DRGs between NCI model and control rats. **E, F** Co-labeling reveals marked enhancement of P2X7R and GFAP signals in the dorsal horn of NCI rats compared with controls, while the CD11b or NeuN levels do no change in P2X7R^+^ cells (scale bar, 100 μm, *n* = 4 per group, *P < 0.05 *vs* CON, two sample *t*-test). **G, H** A438079 (selective P2X7R antagonist) injection slightly suppresses spinal astrocytes compared with NS groups (*n* = 4 rats per group, *P < 0.05 *vs* NS, two sample *t*-test). ***I*** Incubation of spinal slices with A438079 significantly reduces the frequency but not the amplitude of sEPSCs (*n* = 7 for A438079 treatment, *P < 0.05 *vs* Pre, two sample *t*-test). **J** The CRD threshold of NCI rats is significantly increased by 0.5 h after intrathecal injection of A438079 (3 or 10 μg/μL) (*n* = 6 per group, *P < 0.05, **P < 0.01 *vs* NS, two-way ANOVA followed by Tukey’s test). **K** The antinociceptive effect of A438079 persists for 12 h after the final injection of a 7-day daily injection series (*n* = 6 per each, *P <0.05 *vs* Pre, one-way ANOVA followed by Dunnett’s test).

Astrocyte activation factors include *P2X7R, TGFα*, *OSM*, *CNTF*, *IL-6*, and *LIF.* qPCR assays showed that expression of *P2X7R* but not others was strongly up-regulated in the T13-L2 spinal dorsal horn of NCI model rats compared to non-model controls (Fig. 2A). In addition, the P2X7R protein level was increased at 6 and 8 weeks of age in the dorsal horn (Fig. 2B and supplementary Fig. 1F), consistent with the timing of chronic visceral pain in NCI model rats. Notably, we detected no elevation of P2X7R levels in the non-colon-related dorsal horn (Fig. 2C), in the T13-L2 DRG (Fig. 2D), or the T13-L2 dorsal horn at 4 weeks of age in NCI rats (Fig. S1E). Further, the expression of P2X1R, P2X2R, P2X3R, and P2X4R did not differ between NCI and control rats (Fig. S1 A–D). Specifically, immunofluorescence showed that the number of P2X7R^+^ astrocytes was higher in the dorsal horn of NCI rats than in controls, and we found no elevation of P2X7R^+^ microglia or neuronal cells in NCI rats (Fig. 2E, F).

### Inhibition of P2X7R Alleviates Chronic Visceral Pain in NCI Rats

Given our finding that P2X7R specifically accumulated in the dorsal horn astrocytes of NCI rats, we next used a known selective inhibitor of P2X7R (A438079) to study the potential functional impact of P2X7R in the activation of astrocytes and chronic visceral pain [26]. The intrathecal injection of A438079 markedly decreased spinal astrocyte number (Fig. 2G and H). Further, patch-clamp recording revealed that the spinal sEPSC frequency was decreased upon incubation of the slices with A438079; however, the sEPSC amplitude was unchanged (Fig. 2I). Intrathecal injection of A438079 elevated the CRD threshold of NCI rats compared to controls, doing so in a dose- and time-dependent manner. This elevation started within 30 min of A438079 injection (3 or 10 μg/μL) and lasted for ~1 h; no elevation occurred with control saline or 1 μg/μL A438079 injection (Fig. 2J). We noted that daily intrathecal injection of a lower A438079 concentration (3 μg/μL) for a week also increased of CRD threshold, and the longest analgesic effect persisted for 12 h after the final injection on day 7 (Fig. 2K). Collectively, these results show that P2X7R functions in the activation of spinal dorsal horn astrocytes, where it enhances synaptic transmission and promotes the development of NCI-induced chronic visceral pain.

### DNA Hypomethylation of a *p2x7r* CpG Island is increased in NCI Rat Spinal Cord

**Fig. 3:**
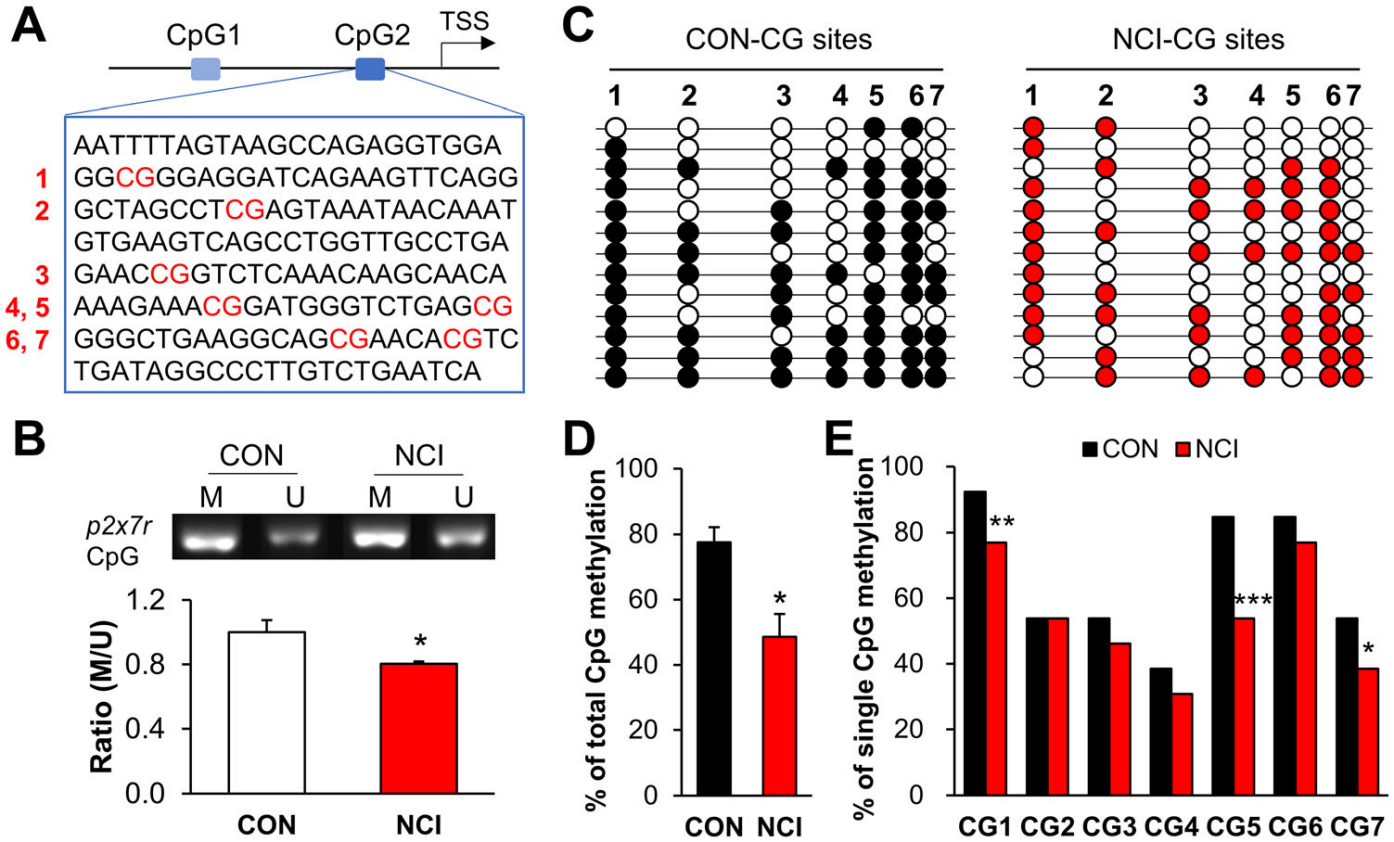
The extent of CpG island methylation at the *p2x7r* locus is decreased in NCI rats. **A** Schematic of the CpG island comprising 7 CG sites in the promoter region of the *p2x7r* locus. **B** Representative methylation-specific PCR results for amplification from the *p2x7r* promoter region using primers for methylated (M) or unmethylated (U) gDNA templates using spinal samples from NCI model and control rats. The bar graph below the gel images shows the ratios of M:U amplification products. NCI rats have a significantly reduced the /U ratio at the CpG island of the *p2x7r* promoter (*n* = 4 per group, *P < 0.05 *vs* CON, two sample *t*-test). **C** Bisulfite sequencing of spinal dorsal horn genes from NCI and control rats. (Black and red solid circle indicate methylated sites). **D** NCI rats exhibited significantly reduced methylation of the *p2x7r* CpG island (*n* = 4 per group, *P < 0.05 *vs* CON, two sample *t*-test). **E** The methylation rates of the CG1, CG5, and CG7 sites were significantly decreased in NCI rats (*n* = 4 per group, *P < 0.05, **P < 0.001, ***P < 0.0001 *vs* CON, two sample *t*-test).

DNA methylation is a fundamental aspect of epigenetics-based gene regulation, and our discovery of elevated P2X7R levels in spinal dorsal horn astrocytes prompted us to examine the DNA methylation status of the *p2x7r* genomic locus. *In silico* analysis using MethPrimer predicted the existence of two putative CpG islands in the *p2x7r* promoter region (Fig. 3A). We conducted both methylation-specific PCR and bisulfite genomic sequence assays and focused on one candidate CpG2 island comprising 7 CG sites because it was located close to the *p2x7r* transcription start site (TSS) (Fig. 3A).

The ratio of methylated to unmethylated CpG sites was significantly lower in NCI samples than in controls, indicating demethylation of this TSS-adjacent CpG island in the *p2x7r* promoter region specifically in the spinal dorsal horn of NCI rats (Fig. 3B). Our bisulfite sequencing further supported this NCI-specific hypomethylation of this CpG2 island (Fig. 3C, D). This analysis also showed significantly reduced methylation at the CG1, CG5, and CG7 sites of the *p2x7r* locus in NCI rats compared with non-model controls (Fig. 3E). Thus, hypomethylation of a TSS-adjacent CpG2 island at the *p2x7r* promoter may contribute to the upregulation of P2X7R in the dorsal horn of NCI rats.

### Up-regulated TET3 in Spinal Astrocytes Contributes to Hypomethylation of a *p2x7r* CpG Island and Chronic Visceral Pain

**Fig. 4:**
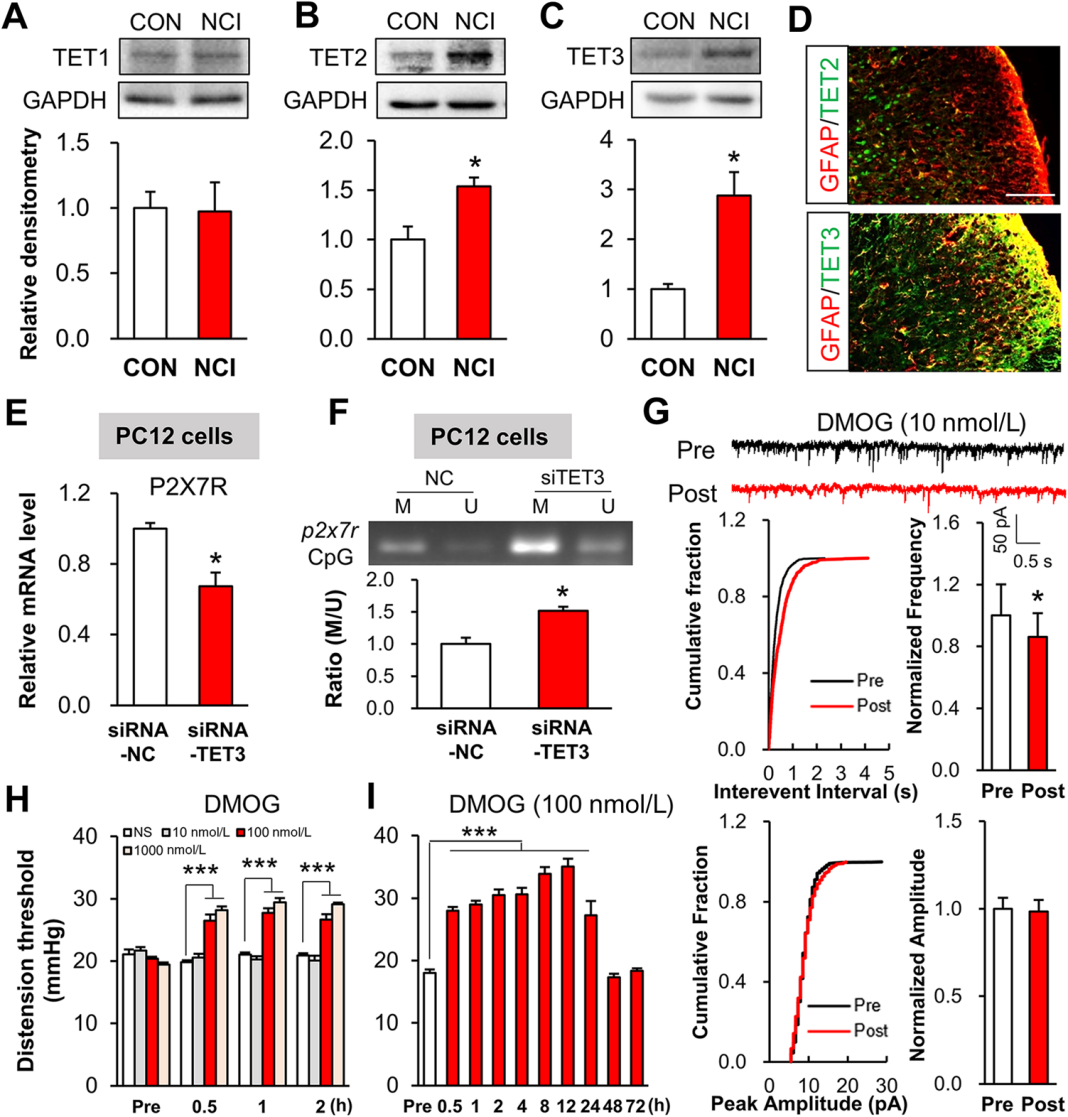
Up-regulated TET3 in spinal astrocytes contributes to chronic visceral pain. **A** The protein expression of TET1 in the dorsal horn does not differ between NCI and control rats (*n* = 4 per group, two sample t-test). **B, C** NCI rats exhibit enhanced accumulation of TET2 and TET3 proteins in the dorsal horn compared to controls (*n* = 4 per group, *P < 0.05 *vs* CON, two sample *t*-test). **D** Immunofluorescence images showing co-localization of GFAP (red) with TET3 (green; lower panel) but not TET2 (green; upper panel) (scale bar, 100 μm). **E** SiRNA-TET3 transfection into cultured PC12 cells (a cell line derived from a pheochromocytoma of the rat adrenal medulla) to achieve knockdown of TET3. The mRNA level of *p2x7r* is significantly decreased after siRNA-TET3-mediated knockdown (*n* = 3 per group, *P < 0.05 *vs* siRNA-NC, two sample *t*-test). **F** SiRNA-TET3 significantly increases the M/U ratio at the TSS-adjacent CpG island of the *p2x7r* promoter (*n* = 3 per group, *P < 0.05 *vs* siRNA-NC, two sample *t*-test). **G** DMOG incubation of spinal slices reduce the sEPSC frequency but does not alter the sEPSC amplitude (*n* = 6 per group, *P < 0.05 *vs* Pre, two sample *t*-test). **H** One intrathecal injection of the nonspecific TET inhibitor DMOG (100 nmol/L or 1000 nmol/L) significantly increases the CRD threshold starting from 0.5 h post-injection and persisting until at least 2 h post-injection (*n* = 6 per group, ***P < 0.0001 *vs* NS, two-way ANOVA followed by Tukey’s *post hoc* test). **I** The CRD threshold is increased from 0.5 to 24 h after daily DMOG injection for a week (*n* = 6 per group, ***P < 0.0001 *vs* NS, one-way ANOVA followed by Dunnett’s test).

DNA methylation is mediated by DNA methyltransferases and demethylases, and ten-eleven translocation proteins (TETs) are well-known DNA hydroxymethylation enzymes. We used western blotting to examine the accumulation of the TET1, TET2, and TET3 proteins in extracts from the spinal dorsal horn of NCI and control rats. Compared to controls, the levels of TET2 and TET3, but not TET1, were up-regulated in the dorsal horn of NCI rats (Fig. 4A–C). However, the mRNA levels of methyltransferases (DNMT1, DNMT3a, and DNMT3b) did not change in the dorsal horn of NCI rats (Fig. S1G). Importantly, immunofluorescence analysis supported the finding that TET3 but not TET2 was expressed in dorsal horn astrocytes (Fig. 4D). In addition, we further examined the role of TET3-induced hypomethylation at a *p2x7r* CpG island *in vitro*. siRNA-TET3 transfection alone into PC12 cells significantly decreased transcription from the *p2x7r* locus (Fig. 4E) while also increasing the extent of DNA methylation at the *p2x7r* CpG island (Fig. 4F).

Given our finding of increased NCI-specific accumulation TET3 in the dorsal horn, we then used the known TET antagonist DMOG to study the functional impact of TET3 on spinal synaptic transmission and visceral pain in NCI rats. Spinal sEPSC frequency was decreased after incubation of spinal slices with DMOG, but there was no change in the sEPSC amplitude (Fig. 4G). Intrathecal injection of DMOG markedly elevated the CRD threshold in NCI model rats, doing so in a dose- and time-dependent manner. The effect started at 30 min and lasted for ~2 h after injection (Fig. 4H). Furthermore, the analgesic effect was evident at 0.5–24 h and returned to normal at 48 h after daily intrathecal injection of DMOG for a week (Fig. 4I). These results together indicate that TET3 functionally contributes to the enhanced spinal synaptic transmission that occurs in NCI model rats.

### GATA1 Contributes to Demethylation of the *p2x7r* CpG Island

**Fig. 5:**
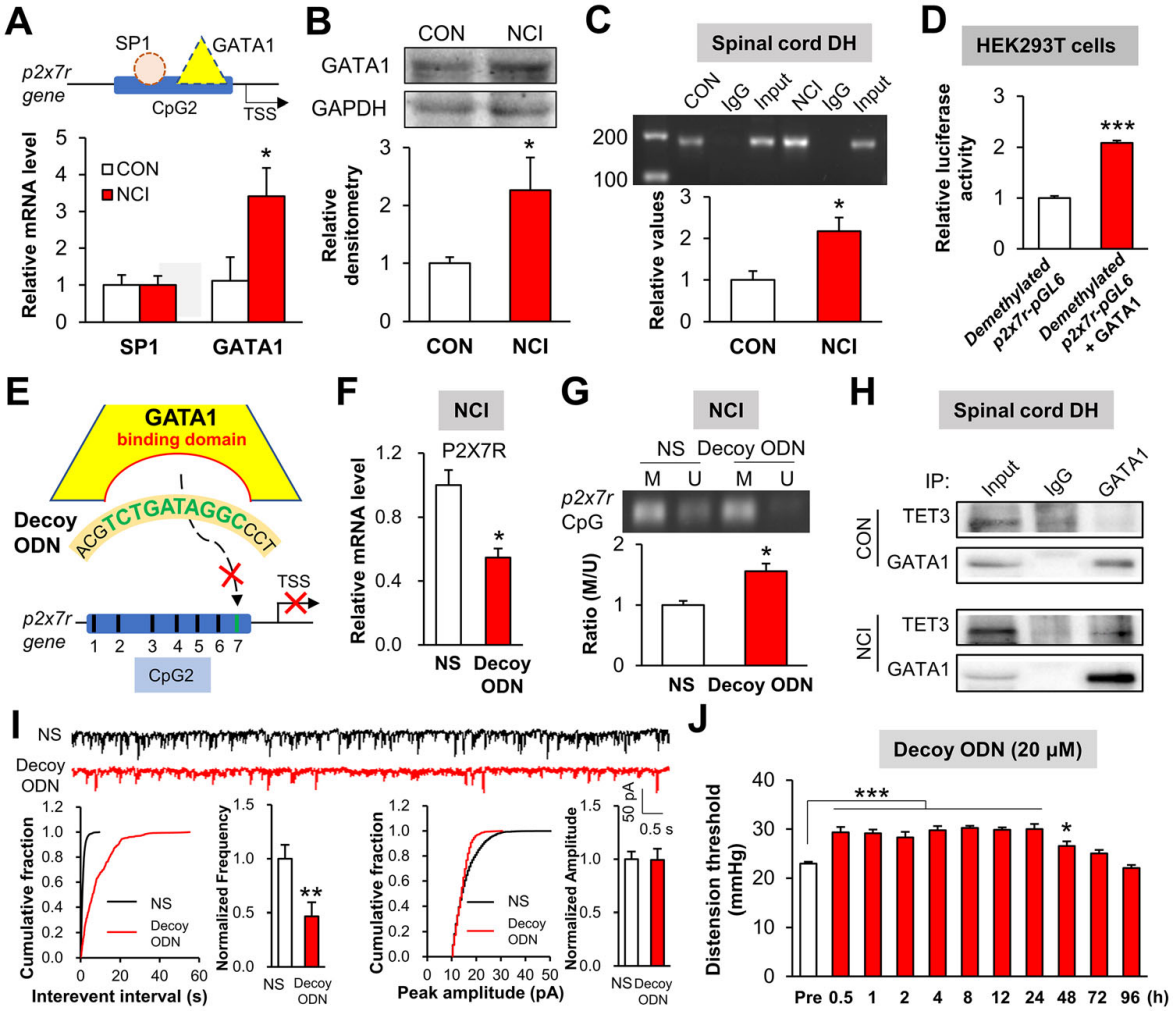
Enhanced binding of GATA1 to the *p2x7r* locus in spinal astrocytes contributes to chronic visceral pain. **A, B** Two transcription factors are predicted to combine with a CpG island in the *p2x7r* promoter (**A**, upper panel). GATA1 expression is dramatically increased at both the protein and mRNA levels in the T13-L2 dorsal horn of NCI rats compared with controls, while the SP1 mRNA level does not change (*n* = 4 per group, *P < 0.05 *vs* CON, two sample *t*-test). **C** ChIP assay showing that NCI significantly enhances the binding of GATA1 to the *p2x7r* promoter in the dorsal horn of NCI rats compared to CON (*n* = 4 per group, *P < 0.05 *vs* CON, two sample *t*-test). **D** Co-transfection of demethylated *p2x7r*-pGL6 and GATA1 plasmid into HEK293T cells enhances the luciferase activity compared with demethylated *p2x7r*-pGL6 transfection alone (*n* = 3 per group, ***P < 0.0001 *vs* demethylated *p2x7r*-pGL6 group, two sample *t*-test). **E** Sequence diagram showing that decoy ODN contains the GATA1 binding site (10 nucleotides) of the *p2x7r* CpG island. **F** The mRNA level of *p2x7r* in the dorsal horn of NCI rats is significantly reduced after intrathecal injection of decoy ODN for a week (*n* = 4 per group, *P < 0.05 *vs* NS, two sample *t*-test). **G** Decoy ODN injection increases the M/U ratio in the *p2x7r* CpG island (*n* = 4 per group, *P < 0.05 vs NS, two sample *t*-test). **H** Co-immunoprecipitation assay revealing a physical interaction between GATA1 and TET3. **I** Decoy treatment of spinal slices from NCI rats reduces the sEPSC frequency but does not alter the sEPSC amplitude (*n* = 10 per group, **P < 0.01 *vs* NS, two sample *t*-test). **J** Daily intrathecal injection of decoy ODN (20 μmol/L in 10 μL) for a week significantly increases the CRD threshold from 0.5 to 48 h (*n* = 6 per group, *P < 0.05, ***P < 0.0001 *vs* Pre, one-way ANOVA followed by Dunnett’s test).

Analysis using the AliBaba2.1 online tool indicated that the transcription factors SP1 and GATA1 are likely to bind at the *p2x7r* CpG island (Fig. 5A, upper panel). Notably, Western blotting and qPCR revealed that expression of GATA1 was greatly increased at both the protein and mRNA level in the dorsal horn of NCI rats compared with controls, while the SP1 mRNA level did not change (Fig. 5A, B). Furthermore, ChIP assays showed modest GATA1–*p2x7r* promoter binding in dorsal horn extracts from control rats, and revealed that GATA1–*p2x7r* promoter binding was elevated in NCI rats (Fig. 5C). To further evaluate the involvement of GATA1 at the *p2x7r* locus, we conducted luciferase reporter gene assays using HEK293T cells. Compared to cells expressing demethylated *p2x7r*-pGL6 alone, there was a dramatic enhancement of luciferase activity in cells co-expressing GATA1 and demethylated *p2x7r*-pGL6 (Fig. 5D).

To investigate the potential impact of GATA1–*p2x7r* promoter binding *in vivo*, we designed and synthesized a decoy ODN containing the specific GATA1 binding sites (Fig. 5E). We noted that decoy ODN treatment decreased the *P2X7R* mRNA level (Fig. 5F). Moreover, given that the predicted GATA1 binding site entails the 7^th^ CG site of the *p2x7r* CpG island, we investigated the methylation status of the *p2x7r* locus, and MSP assays indicated that the extent of demethylation at the *p2x7r* CpG island in the dorsal horn of NCI rats was significantly reduced by treatment with the decoy ODN for 7 days (Fig. 5G). These results indicate that GATA1 binding at the *p2x7r* locus affects the methylation of the *p2x7r* CpG island. However, the means by which GATA1 regulates methylation of the *p2x7r* locus was unknown. Interestingly, it was exciting that co-immunoprecipitation assays revealed the ability of GATA1 to physically interact with TET3 in the dorsal horn; we also found that GATA1–TET3 binding occurred more readily in NCI rats than in non-model controls (Fig. 5H). Collectively, these results suggest that a physical GATA1–TET3 interaction regulates DNA methylation at the CpG island of the *p2x7r* locus.

Furthermore, patch-clamp analysis revealed that the spinal sEPSC frequency was decreased in NCI rats after daily intrathecal injection of decoy ODN for a week; however, the sEPSC amplitude was unchanged (Fig. 5I). In addition, intrathecal injection of decoy ODN remarkably elevated the CRD threshold of NCI rats from 0.5 h to 48 h (Fig. 5J). These results indicate that blockade of GATA1 binding at the *p2x7r* locus attenuates chronic visceral pain in NCI rats through a DNA-methylation-mediated reduction of *P2X7R* transcription.

### Up-regulation of GATA1 in Spinal Astrocytes Contributes to Chronic Visceral Pain

**Fig. 6:**
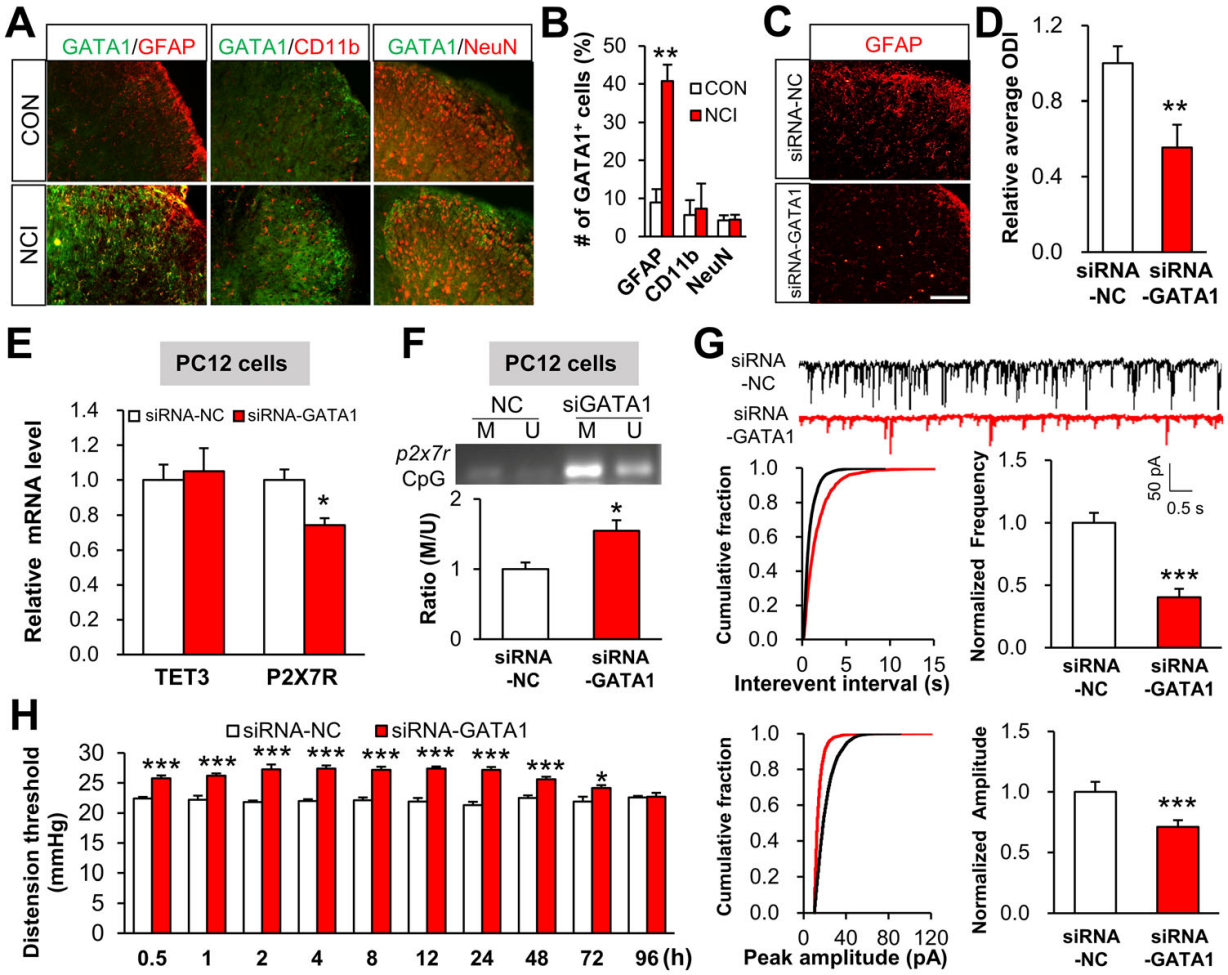
Up-regulation of GATA1 in spinal astrocytes contributes to chronic visceral pain. **A, B** GATA1 (green) is co-expressed with GFAP (red) but not with microglia (red) nor with NeuN (red) (scale bar, 100 μm, *n* = 4 per group, *P < 0.05 *vs* CON, two sample *t*-test). **C, D** SiRNA-GATA1 injection reduces the number of activated astrocytes (scale bar, 100 μm, *n* = 4 per group, **P < 0.01 *vs* siRNA-NC, two sample *t*-test). **E** The mRNA level of *p2x7r* is significantly decreased after siRNA-GATA1-mediated knockdown without affecting TET3 expression (*n* = 3 per group, *P < 0.05 *vs* siRNA-NC, two sample *t*-test). **F** SiRNA-GATA1 significantly increases the M/U ratio at the CpG island adjacent to the TSS of the *p2x7r* promoter (*n* = 3 per group, *P < 0.05 *vs* siRNA-NC, two sample *t*-test). **G** SiRNA-GATA1 (daily intrathecal injection for one week) significantly decreases the frequency and amplitude of sEPSCs (*n* = 10 cells per group, ***P < 0.0001 *vs* siRNA-NC, two sample *t*-test). **H** Intrathecal injection of SiRNA-GATA1 increases the CRD threshold in NCI rats (*n* = 6 per group, *P < 0.05, ***P < 0.001 *vs* siRNA-NC, two-way ANOVA followed by Tukey’s *post hoc* test).

Immunofluorescence analyses showed that GATA1 accumulation was increased in GFAP-positive astrocytes but not CD11b-dyed microglia or NEUN-labeled neurons (Fig. 6A, B). The above findings collectively indicate that up-regulated GATA1 in astrocytes may functionally contribute to chronic visceral pain in NCI rats, so we pursued this by designing siRNA for the specific knockdown of GATA1 expression in NCI rats. Notably, siRNA-mediated knockdown of GATA1 reduced the number of activated astrocytes evaluated by immunofluorescence assays (Fig. 6C and D). Moreover, siRNA-GATA1 transfection of PC12 cells elevated the *P2X7R* mRNA level without affecting TET3 expression (Fig. 6E), which supports the above result that GATA1 binding promotes transcription from the *p2x7r* locus *in vitro* (Fig. 5D). Notably, siRNA-GATA1 transfection significantly decreased the extent of DNA demethylation of the *p2x7r CpG island* (Fig. 6F), further confirming a necessary role of GATA1 in regulating the methylation level of the *p2x7r* locus*.*

Further, patch-clamp recording from spinal cord slices to monitor synaptic transmission showed that NCI rats with GATA1 knockdown had significant reductions in both the frequency and amplitude of sEPSCs compared to scrambled RNA control NCI rats (Fig. 6G). Note that an analgesic effect was evident at 0.5–72 h after administration of siRNA for 7 consecutive days (Fig. 6H). These data together indicate that increased GATA1 in astrocytes of the dorsal horn does contribute to chronic visceral pain in NCI rats.

## Discussion

The need to identify pharmacological compounds that can potently and effectively treat chronic visceral pain has never been more pertinent, with up to 25% of the population reporting this symptom in the clinic. Emerging evidence is building a strong case for the involvement of epigenetic mechanisms in the pathophysiology of many neurological disorders, ranging from chronic pain to psychiatric disturbances [5, 27]. In the present study, we demonstrated that NCI-induced chronic visceral pain is concomitant with an increase in the GATA1-guided DNA demethylation of the *p2x7r* locus in the T13-L2 spinal dorsal horn. Our previous study revealed that NCI enhances the frequency of spinal sEPSCs [25], implying a presynaptic mechanism in NCI-induced visceral pain. In the present study we demonstrated that spinal astrocytes were activated by GATA1/P2X7R signals, suggesting a spinal mechanism of visceral pain. Although it is a different mechanism from the miR-325-5p/CCL2 signals in the DRG described in our recent report, the two mechanisms might play a synergistic role in NCI-induced visceral pain. In addition, we focused on the T13-L2 spinal segments because they are the main projection region from the colon and some other visceral organs [28, 29]. Studies have shown that ~10%–15% of DRG (T13-L2 and L6-S2) neurons are labeled following injection of fluorogold into the descending colon [30], indicating that the same proportion of projections originate from the colon.

There are many examples from studies of endocrine organs showing that GATA factors contribute to the transcriptional regulation of many hormone-encoding genes [31]. Recalling our finding that NCI induced *GATA1* expression and GATA1 protein accumulation specifically in the spinal dorsal horn astrocytes of NCI model rats, and our demonstration of NCI-enhanced binding of GATA1 at the *p2x7r* locus, our study confirms a role for GATA1 in the chronic visceral-hyperalgesia-related up-regulation of P2X7R. In addition, we also measured the mRNA levels of GATA1 in the DRG, anterior cingulate cortex, basolateral amygdala, hippocampus, heart, liver, spleen, lung, and kidney of NCI rats. The GATA1 expression was not altered in the above tissues (data not shown). These results indicated that GATA1 might not play roles in these organs in terms of expression. Note that this previously unknown regulatory mechanism differs from a regulatory mechanism in which a demethylated *p2x3r* locus is modulated by enhanced NF-kB binding [7].

Understanding how GATA factors regulate chronic pain requires the elucidation of epigenetic circuits that control the expression of GATA factor target genes and identification of the GATA factors themselves. Our present study establishes that GATA1 regulates P2X7R expression by affecting DNA methylation at a CpG island of the *p2x7r* locus in spinal dorsal horn astrocytes in NCI model rats. GATA1 knockdown by siRNA significantly blocked the TET3-mediated demethylation of the *p2x7r* promoter. Importantly, demethylation of the *p2x7r* promoter was also prevented when the GATA1 binding site was blocked by decoy ODN. In addition, we showed that crosstalk between transcription factors and demethylases can occur in chronic visceral pain. Specifically, our results support the functional involvement of interactions between GATA1 and the TET3-demethylase at the *p2x7r* promoter. The binding of GATA1 with the *p2x7r* promoter was increased in NCI rats, and IP assays verified the interaction of GATA1 and TET3 protein. While certainly indicative of a regulatory relationship, additional investigations will be needed to fully characterize the temporal and spatial binding sequence underlying the GATA1–TET3 interaction and GATA factor and DNA demethylase cross-talk for chronic visceral-hyperalgesia-related signaling at the *p2x7r* locus. In addition, we found that the application of decoy ODN not only had an analgesic effect in NCI rats but also had an analgesic effect in CFA-induced inflammatory pain and diabetic neuropathic pain (data not shown). Therefore, the GATA1/P2X7R signal pathway might be a critical mechanism in the spinal cord for several pain conditions.

We have previously shown that *p2x3r* DNA demethylation is most likely due to a reduction in DNMT3a/b under diabetic and cancer pain conditions [6, 7]. However, under chronic visceral pain conditions, these two methylation enzymes were not down-regulated, indicating that they are not involved in the demethylation of *p2x7r* in terms of their mRNA expression. The TET proteins are methylcytosine dioxygenases that regulate demethylation by oxidizing 5-methylcytosine to 5-hydroxymethylcytosine (5mhC) and further derivatives [32]. We are unaware of any previous studies reporting significant TET3-mediated DNA demethylation of the *p2x7r* locus, so our finding that the 5hmC-generating TET2-3 proteins are up-regulated in the spinal dorsal horn upon NCI (with TET3 displaying differential up-regulation in GFAP-positive cells) is notable. Currently, it remains unknown whether changes in TET and 5hmC profiles are associated with the pathogenesis of chronic pain. While it is clear that potentially related impact of other types of modification at the same position (*e.g.*, 5-formyl and 5-carboxyl) should be examined, our results support the conclusion that TET3-mediated demethylation of the *p2x7r* locus in spinal dorsal horn astrocytes does regulate nociceptive behavior.

In the central nervous system (CNS), bidirectional astrocyte-neuron signaling boosts fast excitatory synaptic transmission with clear neural circuit and behavioral effects [33]. However, mechanisms underlying the activation of glial cells remain largely unknown. The P2X7R protein has attracted considerable interest as a potential target for various CNS pathologies, including affective and neurodegenerative disorders [34, 35]. In the present study, we provided evidence to support the idea that activation of P2X7R contributes to the activation of spinal astrocytes, since NCI led to up-regulation of P2X7R accumulation and chemical inhibition of P2X7R significantly suppressed both astrocyte activation and spinal synaptic transmission.

P2X7R is expressed in spinal astrocytes, microglia, and neurons under normal conditions throughout early development, although the neuronal expression of the P2X7 receptor has been controversially discussed and contested [19, 36]. We found that NCI caused a significant up-regulation of P2X7R specifically in spinal astrocytes within 6-8 weeks but not 4 weeks, which is consistent with the period of visceral pain reported in our previous study. Unfortunately, we did not detect the expression of P2X7R at 10 weeks since NCI rats did not have visceral pain at 10 weeks. The detailed mechanisms underlying the developmental dynamics of visceral pain need to be further studied. In addition, expression of the other P2X receptors was not changed in the present visceral pain model, further supporting the special effect of P2X7R. This cell-type-specific up-regulation is distinct from previous reports that P2X7R is significantly up-regulated in spinal microglial cells in a rat model of bone cancer pain [37]. This discrepancy could result from differences in the pain models. It is possible that further characterization of pain-model-specific tissue and even cell level effects could help to design and develop specific pain-killers to treat different types of pain (*i.e.*, by targeting not only specific genes but also specific cell types).

Given our finding that a P2X7R antagonist suppressed chronic visceral pain behavior and also inhibited spinal synaptic transmission, our work supports the idea that glial activation might be a common mechanism underlying spinal synaptic plasticity. An implication of this idea is that the ability to restore the normal functions of astrocytes should help to alleviate chronic pain [38]. Spinal glial cells contribute to heightened pain states based on a prolonged release of neurokine signals that sensitize adjacent centrally-projecting neurons [39, 40]. ATP released from activated astrocytes after optogenetic stimulation in the spinal cord induce pain hypersensitivity by inhibiting GABAergic inhibitory interneurons *via* an A1-receptor-dependent pathway [41]. Notably, our present study also demonstrated that astrocytes activated by up-regulated P2X7R enhanced spinal neuronal synaptic transmission in the dorsal horn while we did not elucidate which proalgesic mediators are released by activated astrocytes in NCI model rats. Moreover, we demonstrate that treatment with the astrocyte inhibitor fluorocitrate, with a P2X7R antagonist, and with siGATA1 or decoy ODN in adulthood, normalized the NCI-induced chronic visceral pain. This is, to our knowledge, the first study to demonstrate that early-life inflammation-induced changes in chronic visceral pain processing can be reversed in adulthood *via* inhibition of DNA demethylation pathways in spinal astrocytes.

**Fig. 7:**
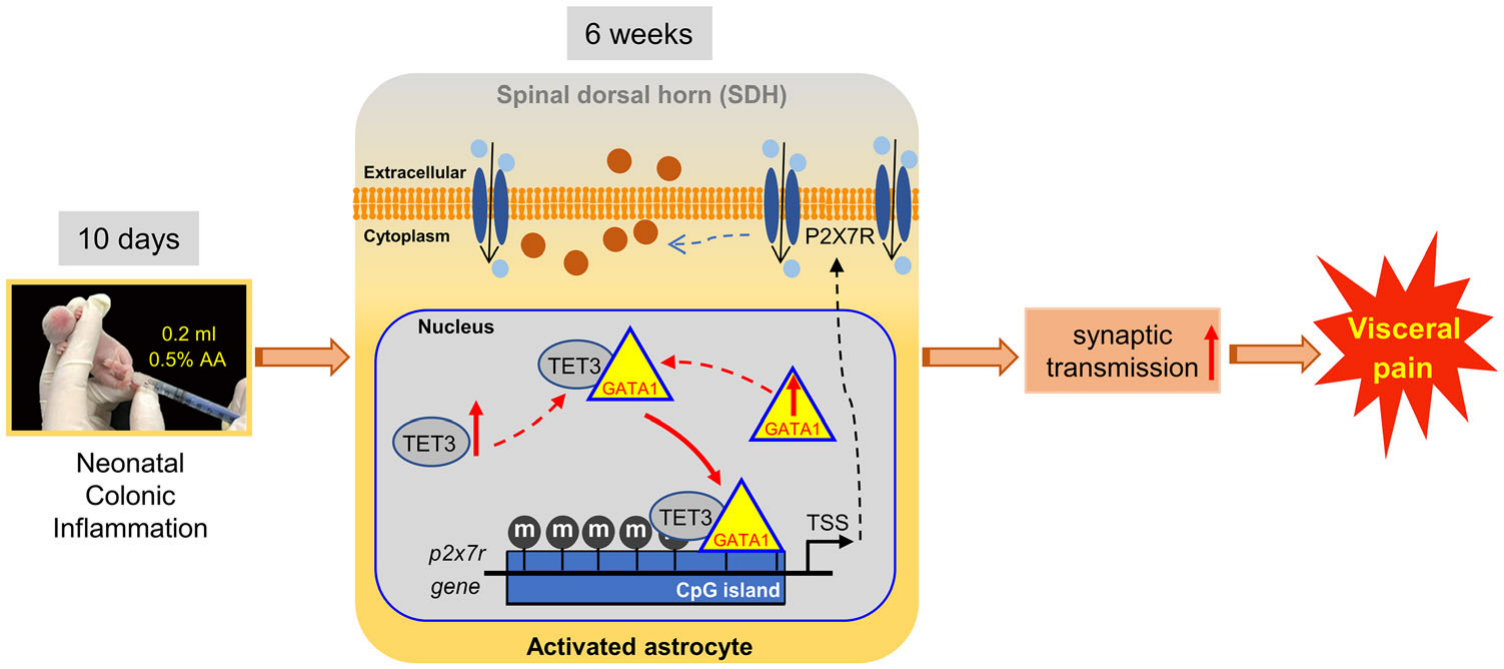
Proposed model for NCI-induced visceral pain. NCI significantly increases P2X7R expression in spinal astrocytes, then activates the astrocytes, and enhances spinal synaptic transmission, thus producing visceral pain. The epigenetic regulation mediated by GATA1 and TET3, leading to DNA demethylation of the *p2x7r* locus*,* together with enhanced binding of GATA1 and *p2x7r*, promotes P2X7R expression in spinal astrocytes.

Taken together, our results add further evidence in support of the roles of epigenetic mechanisms in the pathophysiology of chronic pain. At a minimum, our work supports the conclusion that GATA1-promoted DNA demethylation of the *p2x7r* locus, potentially mediated *via* direct physical interaction with the TET3 demethylase, clearly exerts a regulatory impact on the development of chronic visceral pain in NCI model rats (Fig. 7). Although this mechanism needs to be further confirmed in other animal models (e.g., a stress-induced model of chronic visceral pain), our findings provide a probable potential therapeutic strategy by targeting GATA1 and the *p2x7r* locus binding in the clinical management of patients with gastrointestinal diseases such as IBS.

## Supplementary Information

Below is the link to the electronic supplementary material.

Supplementary file1 (PDF 427 KB)
