# Supplementary material for: Targeting GATA1 and p2x7r Locus Binding in Spinal Astrocytes Suppresses Chronic Visceral Pain by Promoting DNA Demethylation
Source: Neurosci Bull. 2021 Dec 10;38(4):359–72. doi: 10.1007/s12264-021-00799-1 (PMC9068853; doi:10.1007/s12264-021-00799-1)
Supplement: Supplementary file 1 — Supplementary file1 (PDF 427 KB) [file 12264_2021_799_MOESM1_ESM.pdf]

Supplementary figure 1

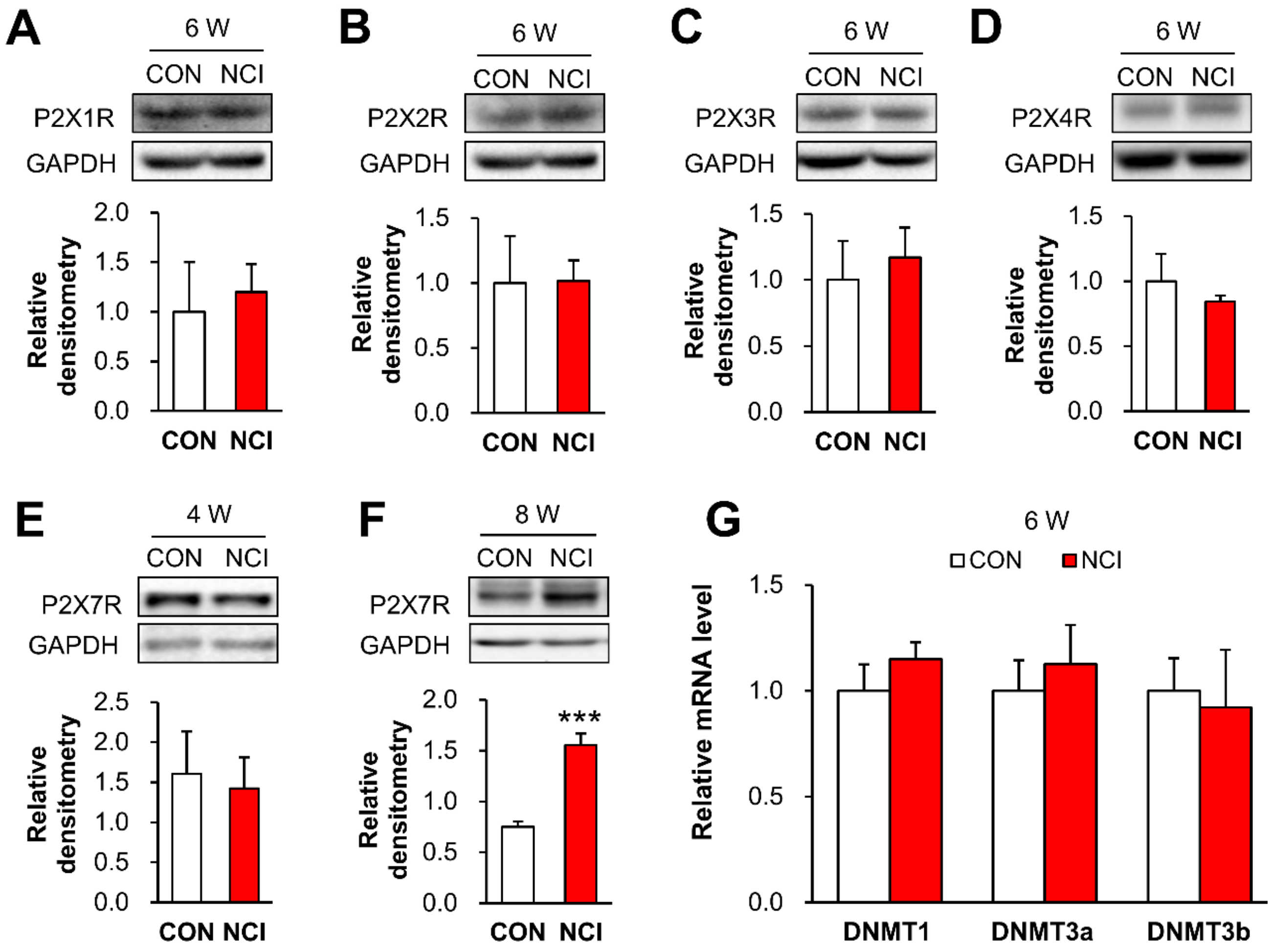

Fig. S1 Expression of P2Xs and DNMTs in T13-L2 spinal dorsal horn. A–D The protein expression of P2X1R, P2X2R, P2X3R, and P2X4R is not altered in the T13-L2 dorsal horn of NCI rats compared with CON rats (*n* = 4 per group, *P* >0.05 *vs* CON, two sample *t*-test). **E** The protein level of P2X7R is not altered in the dorsal horn of NCI rats compared to CON rats at 4 weeks of age (*n* = 4 per group, two sample *t*-test). **F** P2X7R expression is significantly upregulated in NCI rats compared with CON rats at 8 weeks of age (*n* = 4 per group, *P* <0.001 *vs* CON, two sample *t*-test). **G** DNMT1, DNMT3a, and DNMT3b expression are not altered in the dorsal horn of NCI rats compared with CON rats (*n* = 4 per group, *P* >0.05 *vs* CON, two sample *t*-test). W, week.
